# Structure of outer arm dynein molecule in respiratory cilia suggests an alternative mechanism of force generation

**DOI:** 10.1186/2046-2530-4-S1-P78

**Published:** 2015-07-13

**Authors:** H Ueno, H Bui, T Ishikawa, Y Imai, T Yamaguchi, T Ishikawa

**Affiliations:** 1 Aichi University of Education, Kariya, Japan; 2 Paul Scherrer Institute, Villigen PSI, Switzerland; 3 Tohoku University, Sendai, Japan

## Background

Cilia are microtubule (MT)-based organelles that extend from the surface of eukaryotic cells. The ciliary movement is generated by microtubule (MT) sliding with axonemal dynein motors, and plays important roles in cell migration and generation of external fluid flow. Since cilia have diverse roles in many tissues and organs in mammal, defects in ciliary activity causes a number of diseases called ciliopathy.

## Objective

In the ciliary outer dynein arm, two heavy chains produce force during ciliary beating. However, it is still unknown how the ciliary dynein translocate the microtubule using the two different heads.

## Methods

Here, we analyzed the conformational change and its distribution in each dynein head of mouse respiratory cilia by cryo-electron tomography and image processing.

## Results

Most of two heads were in the same form and tightly packed in the non-nucleotide condition, whereas they are dissociated and alternatively moves in the presence of nucleotide. The external side of outer dynein arm shifts toward the neighboring B-tubule and the proximal end of axoneme, while the internal side of head only shifts toward the proximal end. In a significant number of dyneins in the presence of ADP·Vi, two heads overlap each other in the proximal shifting form, indicating that ciliary heterodimeric dynein translocates a microtubule by moving with short steps.

## Conclusion

This indicates that, in contrast to the hand-over-hand motion of cytoplasmic dynein, during ciliary bending axonemal dynein translocates microtubules by moving with short steps.

